# P28 Rezafungin efficacy and safety in immunocompromised patients: sub-analyses of the Phase 3 trial in treatment of candidaemia and invasive candidiasis

**DOI:** 10.1093/jacamr/dlad066.032

**Published:** 2023-06-14

**Authors:** Taylor Sandison, Antonio Ramos-Martinez, Young Keun Kim, Louis Chai, Monica Slavin

**Affiliations:** Cidara Therapeutics Inc., San Diego, CA, USA; Hospital Universitario Puerta de Hierro Majadahonda, Madrid, Spain; Wonju Severance Christian Hospital, Wonju, South Korea; National University Health System, Singapore; Peter MacCallum Cancer Centre and Royal Melbourne Hospital, Melbourne, Australia

## Abstract

**Background:**

Rezafungin (RZF) once weekly (QWk) is a next-generation echinocandin in development for treatment of candidaemia and invasive candidiasis (IC) and for prevention of invasive fungal disease caused by *Candida*, *Aspergillus*, and *Pneumocystis* spp. in BMT.

**Objectives:**

The Phase 3 ReSTORE treatment trial (NCT03667690) demonstrated RZF QWk noninferiority to caspofungin (CAS) QD. This sub-analysis evaluated efficacy and safety outcomes in patients identified as immunocompromised (immuno-c) during the trial.

**Methods:**

As previously described, adults with confirmed candidaemia and/or IC were randomized to RZF QWk (400 mg Wk1 then 200 mg QWk) or CAS QD for ≥14 days (≤4 weeks) with optional oral fluconazole stepdown for CAS. In this sub-analysis, immuno-c patients were those with prior and/or concomitant use of immunosuppressants (e.g. calcineurin inhibitors and corticosteroids) and/or medical history ongoing at screening of neutropenia, BMT, SOT, lymphoma or leukaemia.

**Results:**

Of 187 patients (mITT population), 90 were immuno-c as defined. Results are for those with data available for analyses: Day 14 global cure—Immuno-c: RZF, 51.1% (23/45); CAS, 55.6% (25/45); –No immuno-c: RZF, 66.7% (32/48); CAS 65.3% (32/49). Day 5 mycological eradication—Immuno-c: RZF, 64.4% (29/45); CAS, 51.1% (23/45); no immuno-c: RZF, 72.9% (35/48); CAS, 71.4% (35/49). Among immuno-c, more had ≥1 AE (96.9%) and ≥1 SAE (64.6%) versus no immuno-c (79.0% and 45.0% respectively).

**Conclusions:**

In this sub-analysis of ReSTORE, immune-c status reduced efficacy rates overall but did not change efficacy rate differences between RZF and CAS.

